# Elucidating the Host Interactome of EV-A71 2C Reveals Viral Dependency Factors

**DOI:** 10.3389/fmicb.2019.00636

**Published:** 2019-04-02

**Authors:** Ye Li, Xia Jian, Peiqi Yin, Guofeng Zhu, Leiliang Zhang

**Affiliations:** ^1^NHC Key Laboratory of Systems Biology of Pathogens, Institute of Pathogen Biology, Peking Union Medical College, Chinese Academy of Medical Sciences, Beijing, China; ^2^National Laboratory of Biomacromolecules, CAS Center for Excellence in Biomacromolecules, and Key Laboratory of RNA Biology, Institute of Biophysics, Chinese Academy of Sciences, Beijing, China; ^3^Institute of Basic Medicine, Shandong Academy of Medical Sciences, Jinan, China

**Keywords:** EV-A71, 2C, TRIM4, exportin2, ARFGAP1

## Abstract

Viral protein 2C plays a critical role in EV-A71 replication. The discovery of 2C binding proteins will likely provide potential targets to treat EV-A71 infection. Here, we provide a global proteomic analysis of the human proteins that interact with the EV-A71 2C protein. TRIM4, exportin2, and ARFGAP1 were validated as 2C binding partners. Further functional studies revealed that TRIM4, exportin2, and ARFGAP1 were novel host dependency factors for EV-A71. Moreover, enteroviruses’ 2C family proteins interacted with exportin2 and ARFGAP1. In conclusion, our study provides a cellular interactome of the EV-A71 2C and identifies the proviral roles of TRIM4, exportin2, and ARFGAP1 in EV-A71 infection.

## Introduction

Enterovirus A71 (EV-A71) is one of the major pathogens that leads to hand, foot and mouth disease (HFMD) in young children and infants, and has become a serious threat to global public health (Baggen et al., 2018). EV-A71 outbreaks have been reported particularly in the Asia-Pacific region over the past 15 years. However, current anti-EV-A71 therapy is limited. Development of effective anti-EV-A71 drugs have been hampered by the lack of a detailed understanding of the virus-host interactions that could represent amenable targets for antiviral therapy.

Enterovirus A71 with a positive-stranded RNA genome belongs to the human enterovirus species A of the genus enterovirus within the family *Picornaviridae* (Baggen et al., 2018). The viral genome encodes a single polyprotein precursor which could be proteolytically cleaved to four structural and seven non-structural proteins. The non-structural protein 2C of EV-A71 with 329 amino acids directs replication complexes to cell membranes and contains NTPase and helicase activities (Yuan et al., 2018). Several host factors associated with 2C have been identified. For instance, EV-A71 2C recruited reticulon3 to the viral replication complex (Tang et al., 2007). Coatomer is required for EV-A71 replication and associates with 2C (Wang et al., 2012). 2C binds IKKβ and protein phosphatase 1 to suppress IKKβ phosphorylation (Zheng et al., 2011; Li et al., 2016). By interacting with RelA, 2C inhibited the NF-kB pathway (Du et al., 2015).

Although 2C plays central roles in EV-A71 replication and counteracting the antiviral host defense, there is limited information on how the interaction of 2C with host proteins may contribute to EV-A71 infection. To fill this knowledge gap and advance our understanding of 2C biology, we applied GST pulldown or GFP-Trap immunoprecipitation methods coupled with mass spectrometry analysis to identify the potential binding partners for 2C. Tripartite Motif Protein 4 (TRIM4), exportin2 and ADP Ribosylation Factor GTPase Activating Protein 1 (ARFGAP1) were validated as 2C interacting proteins. Moreover, we demonstrated that TRIM4, exportin2, and ARFGAP1 were required for EV-A71 replication. Our studies will provide the new strategies for the development of host-based antiviral therapy.

## Materials and Methods

### Cells and Reagents

RD and 293T cells were cultured in Dulbecco’s Modified Eagle’s Medium (DMEM, Thermo scientific, Waltham, MA, United States) supplemented with 10% fetal bovine serum (FBS), gentamicin, and glutamine. EV-A71 was cultured in RD cells. The EV-A71 virus used in our study is from the Fuyang strain. QS11 was purchased from Sigma-Aldrich (Piscataway, NJ, United States).

### Antibodies

Mouse antibodies used in this study are listed: anti-actin (Sigma-Aldrich, Piscataway, NJ, United States, catalog no. A2228), anti-dsRNA J2 (English and Scientific Consulting, Hungary), anti-exportin2 (Santa Cruz Biotechnology, Santa Cruz, CA, United States, catalog no. sc-271537), anti-FLAG (Sigma-Aldrich, Piscataway, NJ, United States, catalog no. A2220), anti-GFP (Xuheyuan, Beijing, China, catalog no. XHY038L), anti-Myc (Cell signaling technology, Danvers, MA, United States, catalog no. 2276), anti-HA (Cell signaling technology, Danvers, MA, United States, catalog no. 3724). Rabbit antibodies used in this study are listed: anti-Myc (Cell signaling technology, Danvers, MA, United States, catalog no. 2278) anti-GFP (Xuheyuan, Beijing, China, catalog no. XHY026L), anti-ARFGAP1 (BETHYL, Montgomery, TX, United States, catalog no. A302-029A), anti-TRIM4 (CUSABIO, Wuhan, China, catalog no. CSB-PA866336LA01HU), anti-exportin2 (Abcam, Cambridge, MA, United States, catalog no. ab151546), anti-2C (generated against a peptide from EV-A71 2C [CRDRKSKVRYSVDTVVSELIREYNNRS] conjugated to keyhole limpet hemocyanin [KLH]). Secondary antibodies are HRP-conjugated ECL goat anti-rabbit IgG (Sigma-Aldrich, St. Louis, MO, United States, catalog No. A6154), HRP-conjugated ECL goat anti-mouse IgG (Jackson ImmunoResearch, West Grove, PA, United States, catalog No. A4416), donkey anti-mouse-Alexa Fluor 555, and donkey anti-rabbit-Alexa Fluor 488 (Invitrogen, Carlsbad, CA, United States).

### Plasmids

Constructs encoding for 2C, 2C(126-263), 2C(264-329), ARFGAP1(1-415), ARFGAP1(1-136), and ARFGAP1(137-415) were appended to the carboxyl terminus of glutathione-s-transferase (GST) and were generated using pGEX4T-1 expression plasmids (Amersham Biosciences, Piscataway, NJ, United States). Plasmids expressing TRIM4-Flag and HA-TRIM4 are from Sino Biological (Beijing, China). Plasmid transfected into the cells was performed using FuGENE HD (Promega, Madison, WI, United States) according to the manufacturer instructions.

### Immunofluorescence Microscopy

All procedures were performed at room temperature. Cells in glass coverslips were fixed with 4% formaldehyde in PBS buffer for 5 min. Fixed cells were incubated with blocking solution (PBS containing 10% normal donkey serum) for 5 min and were then incubated with primary antibodies diluted in a permeabilized buffer (0.3% Triton X-100 in PBS containing 10% normal donkey serum) for 1 h. The coverslips were washed three times with blocking solution, followed by incubation with Fluor 488 or Alexa Fluor 555 conjugated secondary antibodies for 1 h. After washing with blocking solution three times, the coverslips were mounted with mounting medium. The cells were imaged with a Leica TCS SP5 microscope (Germany) using a 40× oil immersion lens.

### Immuno-Precipitation Assays

Briefly, cells were lysed in lysis buffer 1 (1% Triton X-100, 50 mM Tris pH 7.4, 150 mM NaCl, protease inhibitor cocktail) or lysis buffer 2 (1% Triton X-100, 50 mM Tris pH 7.4, 90 mM KCl, 2.5 mM MgCl_2_, protease inhibitor cocktail) and incubated with protein A/G beads for 30 min at 4°C to reduce non-specific binding affinity. Cell lysates were then incubated with protein A/G beads pre-bound with 1 μg antibody for 1 h at 4°C. Samples were washed three times with washing buffer (50 mM Tris pH 7.4, 150 mM NaCl, 0.1% Triton X-100), and analyzed by Western blotting.

### GFP-Trap Assays

Cells were lysed with lysis buffer (50 mM Tris pH = 7.5, 150 mM NaCl, 0.5 mM EDTA, 0.5% NP-40, protease inhibitor cocktail) and cell lysates were incubated with GFP-Trap_A beads (ChromoTek, Planegg-Martinsried, Germany) for 1 h at 4°C. The beads were washed three times with wash buffer (50 mM Tris pH = 7.5, 150 mM NaCl, 0.5 mM EDTA) and analyzed by Western blotting.

### GST Pulldown Assay

The expression of the GST fusion protein was induced by 0.5 mM IPTG in *E. coli Rosetta* (DE3) at 37°C for 5 h. Bacteria were lysed with lysis buffer (50 mM Tris pH 6.8, 1 mM EDTA, 100 mM NaCl) and GST fusion proteins were purified using the glutathione-sepharose beads. For the pull-down assay, cell lysates in GST pull-down buffer 1 (1% Triton X-100, 50 mM Tris pH 7.4, 150 mM NaCl, protease inhibitor cocktail) or lysis buffer 2 (1% Triton X-100, 50 mM Tris pH 7.4, 90 mM KCl, 2.5 mM MgCl_2_, protease inhibitor cocktail) were incubated with GST fusion proteins for 1 h at 4°C. Glutathione beads were then pelleted and washed three times with PBS buffer. Samples were analyzed by Western blotting.

### Mass Spectrometry

For mass spectrometry identification, protein complexes pulled down from RD cells were sent to Beijing Protein Innovation. The LC-MS/MS system was composed of a Q Exactive (Thermo scientific, Waltham, MA, United States) mass spectrometer system connected to a Dionex UltiMate 3000. The columns consisted of Thermo 3 μm C18 Acclaim PepMap100 column and Agela Technologies 5 μm C18 Venusil × BPC column. The extract was injected, and the peptides eluted from the column by a 0.1% FA H_2_O /0.1% FA acetonitrile gradient. The nanospray ion source was operated at 1.8 kV.

The data was analyzed using Mascot Software v2.3.0 (Matrix Sciences, London, United Kingdom). The peptide masses were compared with the theoretical peptide masses of all proteins from humans using the SWISS-PROT databases with MASCOT search engine software (Matrix Science).

### Knockdown by siRNA

The siRNAs were transfected into cells using Lipofectamine^TM^ RNAiMAX Transfection Reagent (Invitrogen, Carlsbad, CA, United States). The siRNAs used for TRIM4, exportin2, ARFGAP1 knockdown were from GenePharma (Shanghai, China) and were as follows: GAAGUUGAGAGUAGAGAUATT (TRIM4 #1), GAGAUUGAACAAAGAAGAATT (TRIM4 #2),GAAGACAGUGUGCCAGAUATT (TRIM4 #3), GCATGGAATTACAAAGCAAA (exportin2 #1), GACGGUAUCAAAUAUAUUATT (exportin2 #2), GGAACUCAGCGAUGCAAAUTT(exportin2 #3), ACAUUGAGCUUGAGAAGAU (ARFGAP1 #1), and ACAGGAGAAGUACAACAGCAGA (ARFGAP1 #2).

### Quantitative PCR (qPCR)

Total cellular and viral RNA was isolated using RNeasy Mini columns (QIAGEN) and reverse transcribed by random priming with the High Capacity cDNA Reverse Transcription Kit (Applied Biosystems; Foster City, CA, United States), then quantitated by qPCR using the DyNAmo HS SYBR Green qPCR Kit (Finnzyme; Espoo, Finland). Sequences of primers used in qPCR were as follows: The forward and reverse primers for EV-A71 were 5′- GCAGCCCAAAAGAACTTCAC-3′ and5′-ATTTCAGCAGCTTGGAGTGC-3′; the forward and reverse primers for GAPDH were 5′-ACCTTCCCCATGGTGTCTGA-3′ and 5′-GCTCCTCCTGTTCGACAGTCA-3′; the forward and reverse primers for exportin2 were 5′- CGCACCGTTTGTTGAGATTC-3′ and 5′-TGATGAGAGTAGGGATGTAGGG-3′; the forward and reverse primers for TRIM4 were 5′-ATGCTAAAGCGATTCCAAGTG-3′ and 5′-CAAGAACTGGCTGATGCTGTAT-3′; the forward and reverse primers for ARFGAP1 were 5′- GCGCATCCTCATTGCAG-3′ and 5′-CTTCCTGGTTCTTGGGCTG-3′.

### Virus Entry Assay

The EV-A71 virus entry was measured as previously described (Xu et al., 2016). Briefly, RD cells were washed with cold PBS, followed by incubation with viruses on ice. Cells were then incubated with viruses for 30 min on ice. The cells were washed with cold PBS and then incubated at 37°C for 1 h to allow virus entry before being treated with trypsin to remove any viruses that bound to the cell surface. Total RNA was isolated, and the levels of viral RNA were determined by qPCR.

### Plaque Assay

Virus titers were measured through a plaque assay. RD cells were seeded into 6-well plates. The EV-A71 virus was diluted with 10-fold serial dilutions and then incubated with RD cells for 2 h at 37°C. The cells were overlaid with DMEM (10% FBS) containing 1% agarose. Three days later, the cells were stained with Crystal violet and the viral plaques were counted.

### Cell Viability Assay

Cell viability was assessed using the Cell Titer-Glo Luminescent Cell Viability Assay Kit (Promega, Madison, WI, United States) according to the manufacturer’s protocol.

### Statistics

Statistically significant differences were assessed using the paired Student’s *t*-test from GraphPad Prism 5 (GraphPad Software Inc., La Jolla, CA, United States). Data represent the averages from at least three independent experiments ± (standard deviation) SD, unless stated otherwise. ns, no significance; ^∗^*P* < 0.05; ^∗∗^*P* < 0.001; ^∗∗∗^*P* < 0.0001.

## Results

### Identification of 2C Interacting Proteins

**FIGURE 1:**
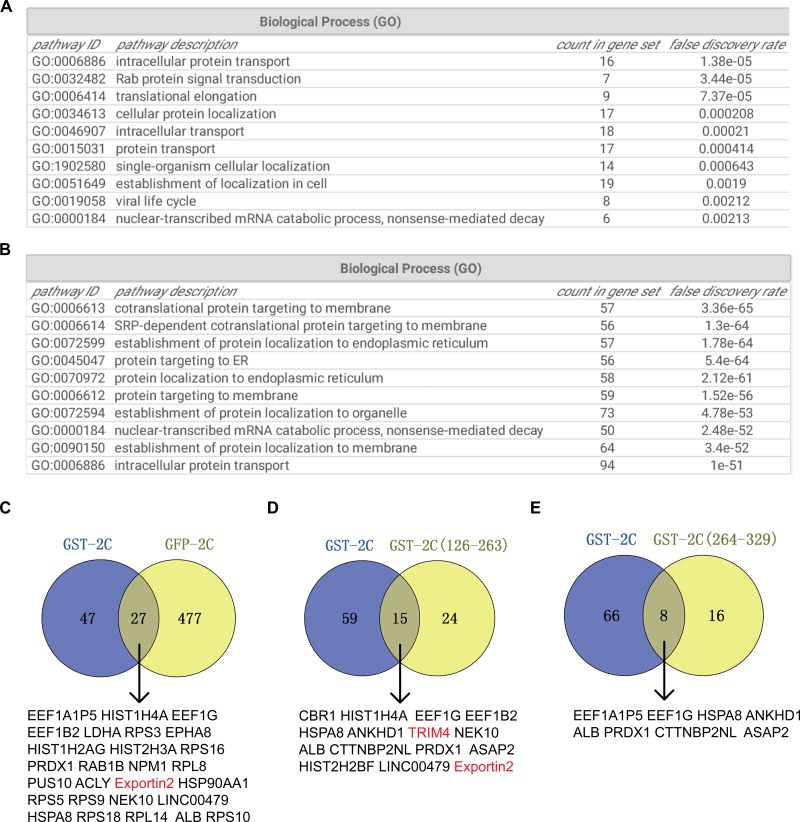
Host interactome analysis of the EV-A71 2C protein. **(A)** Gene Ontology (GO)-term analysis of potential binding partners for GST-2C. **(B)** GO-term analysis of potential binding partners for GFP-2C. **(C)** Venn diagram showing the overlap of potential binding partners for GST-2C and GFP-2C. **(D)** Venn diagram showing the overlap of potential binding partners for GST-2C and GST-2C(126-263). **(E)** Venn diagram showing the overlap of potential binding partners for GST-2C and GST-2C(264-329).

To elucidate the interactome of EV-A71 2C, we performed GST pull-down coupled with mass spectrometry. Constructs encoding for GST-2C, GST-2C(1-125), GST-2C(126-263), or GST-2C(264-329) were appended to the carboxyl terminus of GST and were generated using pGEX4T-1 expression plasmids. GST-2C(1-125) was not soluble in the bacteria and thus was removed from the study. The GST fusion protein or GST was accumulated onto glutathione beads and incubated with cell lysates for pull-down assay. We also applied the GFP-Trap to precipitate GFP-2C or GFP associated proteins. For mass spectrometry identification, the protein complexes pulled down were sent to Beijing Protein Innovation. To analyze the mass spectrometry results, we excluded the proteins that showed up in the control GST or the control GFP-Trap condition. We identified 74 hits for GST-2C (Supplementary Table 1), 39 hits for GST-2C(126-263) (Supplementary Table 2), 24 hits for GST-2C(264-329) (Supplementary Table 3), and 504 hits for GFP-2C (Supplementary Table 4). The top ten biological processes in the Gene Ontology (GO)-term analysis of GST-2C binding partners is shown in Figure 1A. The top ten biological processes in the GO-term analysis of GFP-2C binding partners are shown in Figure 1B. There are 27 overlapped hits for GST-2C and GFP-2C (Figure 1C), 15 overlapped binding proteins for GST-2C and GST-2C(126-263) (Figure 1D), and eight overlapped binding proteins for GST-2C and GST-2C(264-329) (Figure 1E). In the overlapped binding proteins, most were transcription factors, protein chaperons, ribosomal proteins, or histones, which were sticky. Thus, we picked up TRIM4 and exportin2 to investigate their role in the viral life cycle. Key components of COPI including coatomer and ARF1 have been identified as host factors for EV-A71 (Wang et al., 2014), thus we also chose another COPI component ARFGAP1, from the hit for GFP-2C, for further study.

### Confirmation of the Interaction Between TRIM4 and 2C

**FIGURE 2:**
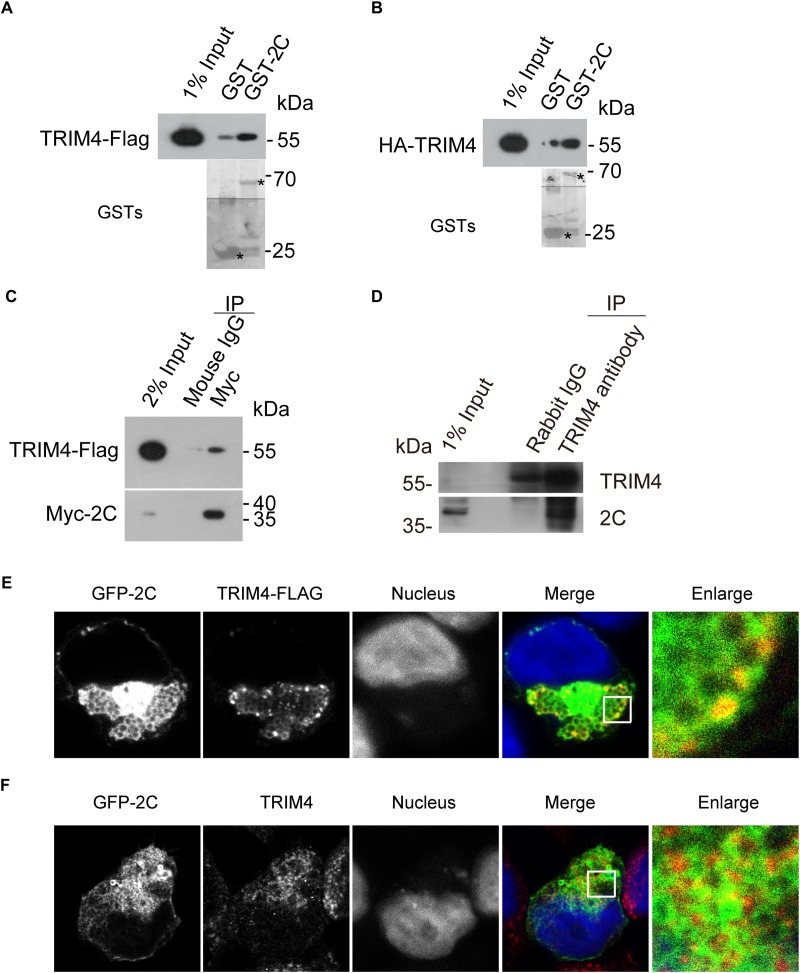
Validation of the interaction between TRIM4 and 2C. **(A)** Validation of the interaction between the GST-2C and TRIM4-Flag. Cell lysates from 293T cells transfected with the plasmid expressing TRIM4-Flag were pulled down by GST–2C or GST followed by Western blotting. ^∗^ indicated GST or GST fustion proteins. **(B)** Validation of the interaction between the GST-2C and HA-TRIM4. Cell lysates from 293T cells transfected with the plasmid expressing HA-TRIM4 were pulled down by GST–2C or GST followed by Western blotting. ^∗^ indicated GST or GST fustion proteins. **(C)** Immunoblots showing the association of 2C with TRIM4 in transfected 293T cells by a co-immunoprecipitation assay. 293T cells were transfected with TRIM4-Flag and Myc-2C for 48 h. Cell lysates were incubated with mouse anti-Flag antibody or mouse IgG, and co-immunoprecipitated proteins were subjected to Western blotting for analysis. **(D)** Validation of the interaction between the 2C and TRIM4 during EV-A71 replication. Cell lysates from RD cells infected with EV-A71 were immunoprecipitated by rabbit TRIM4 antibody or control rabbit IgG followed by Western blotting. **(E)** Colocalization of GFP-2C with TRIM4-Flag in 293T cells. 293T cells transfected with GFP-2C and TRIM4-Flag were fixed by PFA and immunofluorescently labeled for Flag. DAPI staining indicates the nucleus (blue). **(F)** Colocalization of GFP-2C with TRIM4 in 293T cells. 293T cells transfected with GFP-2C were fixed by PFA and immunofluorescently labeled for TRIM4. DAPI staining indicates the nucleus (blue).

To validate the interaction between TRIM4 and 2C, 293T cells were transiently transfected with constructs expressing Flag-tagged TRIM4 or HA-tagged TRIM4. Cell lysates were incubated with Glutathione-Sepharose beads containing GST or GST-2C. We found that GST-2C but not GST was able to pull down TRIM4 (Figure 2A,B).

To further confirm the interaction between TRIM4 and 2C, 293T cells were transiently transfected with constructs expressing Flag-tagged TRIM4 and Myc-tagged 2C. As shown in Figure 2C, mouse Myc antibody but not control mouse IgG was able to precipitate TRIM4. To validate the interaction between TRIM4 and 2C during virus infection, lysates from RD cells infected with EV-A71 were incubated with rabbit TRIM4 antibody or control rabbit IgG. As shown in Figure 2D, TRIM4 associated with 2C during EV-A71 infection.

The specific interaction of EV-A71 2C with TRIM4 prompted us to examine whether these proteins colocalized by immunofluorescence microscopy. First, 293T cells transfected constructs expressing GFP-2C and TRIM4-Flag were subjected to immunofluorescence staining, using rabbit anti-Flag antibody. As shown in Figure 2E, TRIM4 colocalized with the EV-A71 2C protein. Next, 293T cells transfected constructs expressing GFP-2C were subjected to immunofluorescence staining, using rabbit anti-TRIM4 antibody. Endogenous TRIM4 were also colocalized with GFP-2C (Figure 2F).

### The Roles of TRIM4 in EV-A71 Replication

**FIGURE 3:**
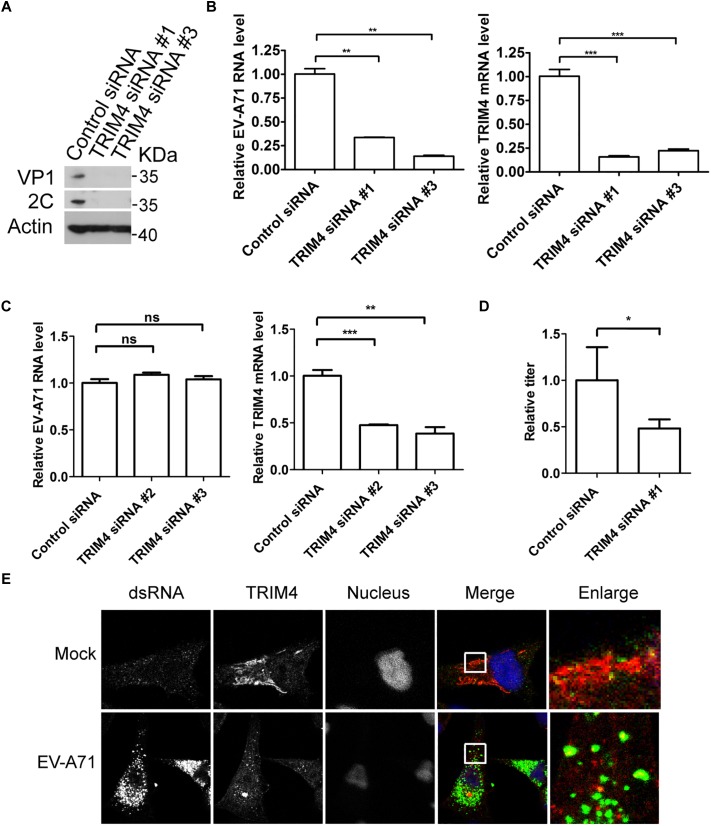
TRIM4 is required for EV-A71 replication. **(A)** RD cells were treated with siRNA targeting TRIM4 or control siRNA for 72 h and infected with EV-A71 (MOI = 1) for another 8 h. The cell lysates were analyzed by immunoblotting with the indicated antibodies. **(B)** RD cells were treated with siRNAs targeting TRIM4 or control siRNA for 72 h and then infected with EV-A71 (MOI = 1) for 6 h. Relative levels of EV-A71 RNA and TRIM4 mRNA were quantified by qPCR and normalized to GAPDH mRNA. Data represent the averages from at least three independent experiments ± SD. ^∗∗^, *P* < 0.001; ^∗∗∗^, *P* < 0.0001. **(C)** RD cells were treated with siRNA targeting TRIM4 or control siRNA for 72 h. Cells were then incubated with viruses for 30 min on ice. The cells were washed with cold PBS and then incubated at 37°C for 1 h to allow virus entry before treated with trypsin to remove any viruses that bound to cell surface. ns, no significance; ^∗∗^, *P* < 0.001; ^∗∗∗^, *P* < 0.0001. **(D)** RD cells were treated with siRNA targeting TRIM4 or control siRNA for 72 h and infected with EV-A71 (MOI = 1) for another 20 h. Virus from the supernatant was tittered. ^∗^, *P* < 0.05. **(E)** RD cells were infected with EV-A71 (MOI = 1) or mock infected for 8 h and then stained with antibodies against dsRNA and TRIM4. DAPI staining indicates the nucleus (blue).

To access the role of TRIM4 in the EV-A71 life cycle, we applied a siRNA strategy to silence TRIM4. The EV-A71 2C and VP1 proteins were reduced when TRIM4 was knocked down by two siRNA 3 days before EV-A71 infection (Figure 3A). TRIM4 depletion also reduced EV-A71 viral RNA replication assessed by QPCR (Figure 3B). However, silencing TRIM4 did not change virus entry (Figure 3C). Furthermore, the EV-A71 virus titer was reduced by siRNA against TRIM4 (Figure 3D). Altogether, TRIM4 is required for EV-A71 replication.

Next, we examined the localization of TRIM4 in EV-A71 infected cells. As shown in Figure 3E, TRIM4 colocalized with viral dsRNA, indicating that TRIM4 might be involved in viral replication.

### Confirmation of the Interaction Between Exportin2 and 2C

**FIGURE 4:**
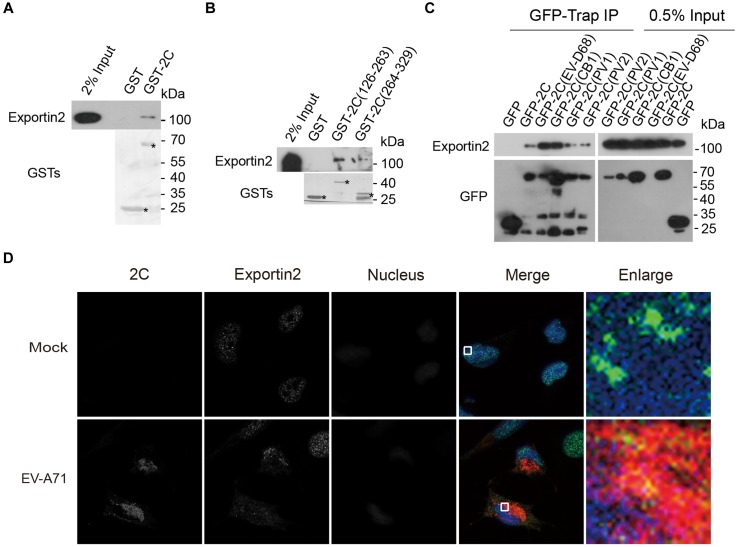
Validation of the interaction between exportin2 and 2C. **(A)** Validation of the interaction between the GST-2C and exportin2. Cell lysates from 293T cells were pulled down by GST-2C or GST followed by Western blotting. ^∗^ indicated GST or GST fustion proteins. **(B)** Identification of the interaction between the GST-2C(126-263) and exportin2. Cell lysates from 293T cells were pulled down by GST-2C(126-263), GST-2C(264-329), or GST followed by Western blotting. ^∗^ indicated GST or GST fustion proteins. **(C)** 293T cells were transfected with constructs encoding GFP tagged 2C proteins from PV1, PV2, CB1, EV-D68, or EV-A71 and then immunoprecipitated with GFP-Trap. Samples were analyzed with Western blotting. **(D)** Colocalization of 2C with exportin2 in RD cells. RD cells were infected with EV-A71 (MOI = 1) or mock infected for 8 h and then stained with antibodies against 2C and exportin2. DAPI staining indicates the nucleus (blue).

To validate the interaction between exportin2 and 2C, lysates from 293T cells were incubated with GST and GST-2C. GST-2C but not GST was able to pull down exportin2 (Figure 4A). To map the region in 2C for the association with exportin2, 293T cell lysates were incubated with GST, GST-2C(126-263), and GST-2C(264-329). As shown in Figure 4B, GST-2C(126-263) but not GST-2C(264-329) was able to interact with exportin2.

Next, we accessed whether 2C-exportin2 interaction is conserved in enteroviruses. Constructs encoding the GFP tagged 2C proteins from poliovirus type I (PV1), poliovirus type II (PV2), coxsackievirus B1 (CB1), enterovirus D68 (EV-D68), and EV-A71 were transfected into 293T cells. We performed the GFP-Trap experiment to investigate whether enteroviruses’ 2C proteins could bind exportin2. All enteroviruses’ 2C proteins we tested were able to interact with exportin2 (Figure 4C), suggesting that those interactions are conserved across enteroviruses. Next, we examined whether 2C and exportin2 colocalized by immunofluorescence microscopy. RD cells infected with EV-A71 (MOI = 1) or mock infected for 8 h were subjected to immunofluorescence staining using rabbit anti-2C antibody and mouse anti-exportin2 antibody. As shown in Figure 4D, endogenous exportin2 colocalized with 2C in EV-A71-infected cells, indicating that exportin2 could associate with 2C in the same intracellular area.

### The Roles of Exportin2 in EV-A71 Replication

**FIGURE 5:**
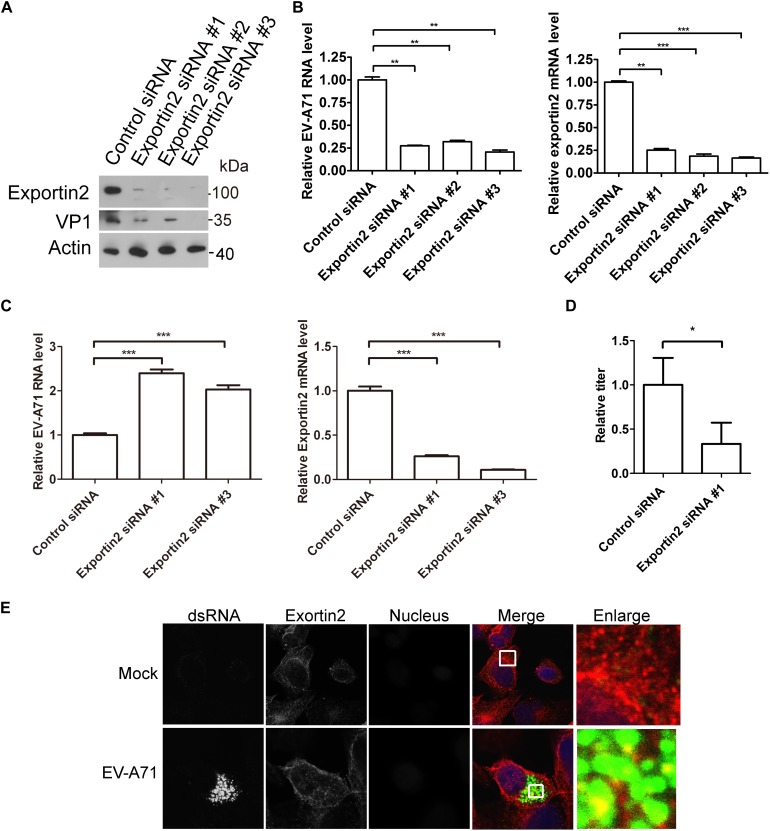
Exportin2 is required for EV-A71 replication. **(A)** RD cells were treated with siRNA targeting exportin2 or control siRNA for 72 h and infected with EV-A71 (MOI = 1) for another 8 h. The cell lysates were analyzed by immunoblotting with the indicated antibodies. **(B)** RD cells were treated with siRNAs targeting exportin2 or control siRNA for 72 h and then infected with EV-A71 (MOI = 1) for 6 h. Relative levels of EV-A71 RNA and exportin2 mRNA were quantified by qPCR and normalized to GAPDH mRNA. Data represent the averages from at least three independent experiments ± SD. ^∗∗^, *P* < 0.001; ^∗∗∗^, *P* < 0.0001. **(C)** RD cells were treated with siRNA targeting exportin2 or control siRNA for 72 h. Cells were then incubated with viruses for 30 min on ice. The cells were washed with cold PBS and then incubated at 37°C for 1 h to allow virus entry before treated with trypsin to remove any viruses that bound to cell surface. ^∗∗∗^, *P* < 0.0001. **(D)** RD cells were treated with siRNA targeting exportin2 or control siRNA for 72 h and infected with EV-A71 (MOI = 1) for another 20 h. Virus from the supernatant was tittered. ^∗^, *P* < 0.05. **(E)** RD cells were infected with EV-A71 (MOI = 1) or mock infected for 8 h and then stained with antibodies against dsRNA and exportin2. DAPI staining indicates the nucleus (blue).

To access the potential role of exportin2 in the EV-A71 life cycle, we applied the siRNA strategy to silencing exportin2. The EV-A71 VP1 protein was decreased when exportin2 was knocked down by three siRNA 3 days before EV-A71 infection (Figure 5A). Exportin2 depletion also reduced EV-A71 viral RNA replication assessed by QPCR (Figure 5B). Interestingly, silencing exportin2 increased virus entry (Figure 5C). Moreover, the EV-A71 virus titer was reduced by siRNA against exportin2 (Figure 5D). Altogether, exportin2 is a host dependency factor for EV-A71.

Next, we examined the localization of exportin2 in EV-A71 infected cells. As shown in Figure 5E, exportin2 partially colocalized with viral dsRNA, indicating that exportin2 might be involved in viral replication.

### Validation of the Interaction Between ARFGAP1 and 2C

**FIGURE 6:**
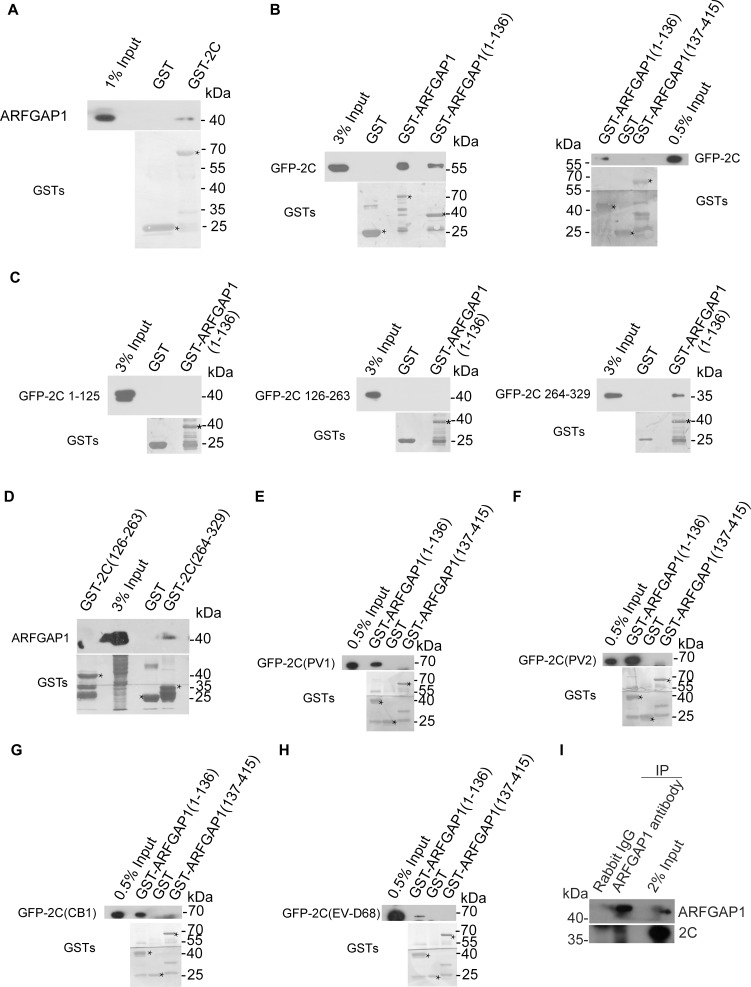
Validation of the interaction between ARFGAP1 and 2C. **(A)** Validation of the interaction between the GST-2C and ARFGAP1. Cell lysates from 293T cells were pulled down by GST–2C or GST followed by Western blotting. ^∗^ indicated GST or GST fustion proteins. **(B)** Identification of the interaction between the GFP-2C and GST-ARFGAP1(1-136). Cell lysates from 293T cells transfected with GFP-2C were pulled down by GST-ARFGAP1(1-136), GST-ARFGAP1(137-415), or GST followed by Western blotting. ^∗^ indicated GST or GST fustion proteins. **(C)** Identification of the interaction between the GST-2C(264-329) and GST-ARFGAP1(1-136). Cell lysates from 293T cells transfected with GFP-2C(1-125), GFP-2C(126-263), or GFP-2C(264-329) were pulled down by GST-ARFGAP1(1-136) or GST followed by Western blotting. ^∗^ indicated GST or GST fustion proteins. **(D)** Confirmation of the interaction between the GST-2C(264-329) and ARFGAP1(1-136). Cell lysates from 293T cells were pulled down by GST-2C(126-263), GST-2C(264-329), or GST followed by Western blotting. **(E–H)** Constructs encoding GFP tagged 2C proteins from PV1 **(E)**, PV2 **(F)**, CB1 **(G)**, and EV-D68 **(H)** were transfected into 293T cells. Cell lysates were pulled down by GST-ARFGAP1(1-136), GST-ARFGAP1(137-415), or GST followed by Western blotting. ^∗^ indicated GST or GST fustion proteins. **(I)** Validation of the interaction between the 2C and ARFGAP1 during EV-A71 replication. Cell lysates from RD cells infected with EV-A71 were immunoprecipitated by rabbit ARFGAP1 antibody or control rabbit IgG followed by Western blotting.

To validate the interaction between ARFGAP1 and 2C, lysates from 293T cells were incubated with GST and GST-2C. GST-2C but not GST was able to pull down ARFGAP1 (Figure 6A). To identify the critical region of ARFGAP1 for association with 2C, lysates from 293T cells transfected with constructs expressing GFP-2C were incubated with GST, GST-ARFGAP1(1-136), or GST-ARFGAP1(137-415). GST-ARFGAP1(1-136) but not GST-ARFGAP1(137-415) was able to pull down ARFGAP1 (Figure 6B). To identify the critical region of 2C for association with ARFGAP1, 293T cells were transiently transfected with constructs expressing GFP-tagged 2C(1-125), 2C(126-263), or 2C(264-329). Lysates were incubated with GST, GST-ARFGAP1(1-136). 2C(264-329) but not 2C(1-125) nor 2C(126-263) was able to pull down ARFGAP1(1-136) (Figure 6C). To further confirm the interaction between ARFGAP1 and 2C(264-329), lysates from RD cells were incubated with GST, GST-2C(126-263), or GST-2C(264-329). GST-2C(264-329) but not GST-2C(126-263) was able to pull down ARFGAP1 (Figure 6D).

Next, we investigated whether 2C-ARFGAP1 interaction is conserved in enteroviruses. Constructs encoding GFP tagged 2C proteins from PV1, PV2, CB1, EV-D68, and EV-A71 were transfected into 293T cells. We performed the GST pull-down experiment to investigate whether enteroviruses’ 2C proteins were able to bind ARFGAP1. All enteroviruses’ 2C proteins in our study associated with ARFGAP1 (Figure 6E–H), indicating that 2C-ARFGAP1 interaction is conserved across enteroviruses.

To confirm the interaction between ARFGAP1 and 2C during viral replication, lysates from RD cells infected with EV-A71 were immunoprecipitated by rabbit ARFGAP antibody or control rabbit IgG. As shown in Figure 6I, 2C associated with ARFGAP1 during EV-A71 infection.

### The Roles of ARFGAP1 in EV-A71 Replication

**FIGURE 7:**
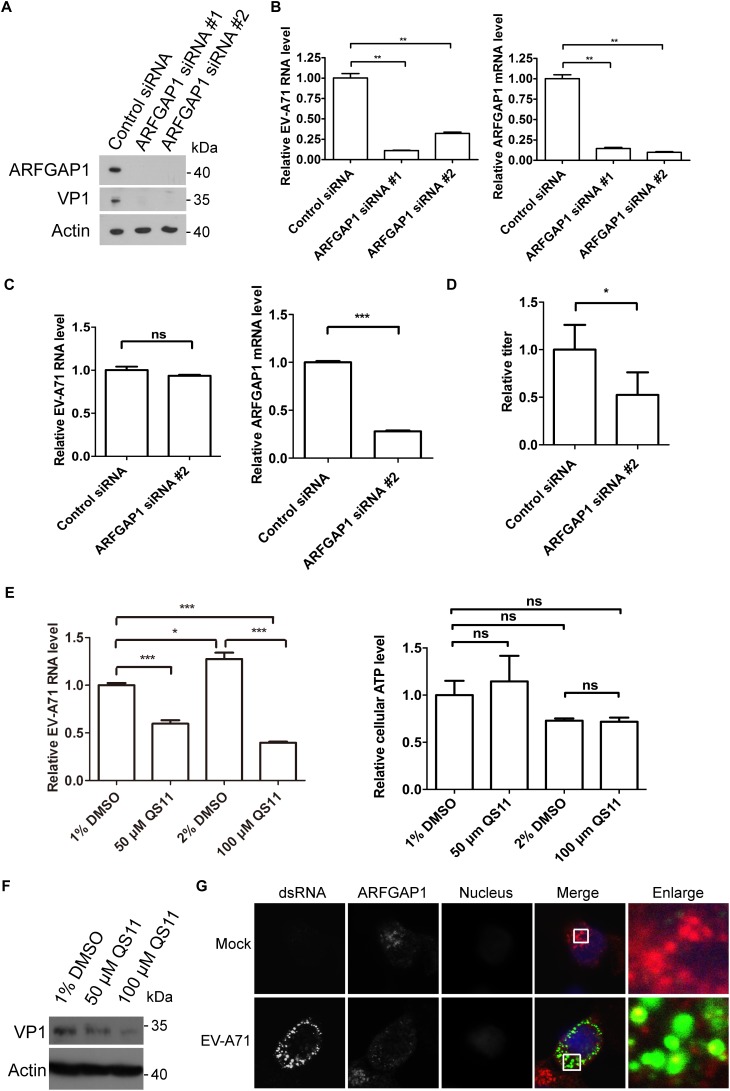
ARFGAP1 is required for EV-A71 replication. **(A)** RD cells were treated with siRNA targeting ARFGAP1 or control siRNA for 72 h and infected with EV-A71 (MOI = 1) for another 8 h. The cell lysates were analyzed by immunoblotting with the indicated antibodies. **(B)** RD cells were treated with siRNAs targeting ARFGAP1 or control siRNA for 72 h and then infected with EV-A71 (MOI = 1) for 6 h. Relative levels of EV-A71 RNA and ARFGAP1 mRNA were quantified by qPCR and normalized to GAPDH mRNA. Data represent the averages from at least three independent experiments ± SD. ^∗∗^, *P* < 0.001. **(C)** RD cells were treated with siRNA targeting ARFGAP1 or control siRNA for 72 h. Cells were then incubated with viruses for 30 min on ice. The cells were washed with cold PBS and then incubated at 37°C for 1 h to allow virus entry before treated with trypsin to remove any viruses that bound to cell surface. ns, no significance; ^∗∗∗^, *P* < 0.0001. **(D)** RD cells were treated with siRNA targeting ARFGAP1 or control siRNA for 72 h and infected with EV-A71 (MOI = 1) for another 20 h. Virus from the supernatant was tittered. ^∗^, *P* < 0.05. **(E)** RD cells were infected with EV-A71 (MOI = 1) for 1 h and then treated with QS11 or DMSO for 5 h. Total RNA was isolated, and the levels of viral RNA were determined by qPCR. The cell viability was measured by cellular ATP level. ns, no significance; ^∗^, *P* < 0.01; ^∗∗∗^, *P* < 0.0001. **(F)** RD cells were infected with EV-A71 (MOI = 1) for 1 h and then treated with QS11 or DMSO for 7 h. The cell lysates were analyzed by immunoblotting with the indicated antibodies. **(G)** RD cells were infected with EV-A71 (MOI = 1) or mock infected for 8 h and then stained with mouse dsRNA antibody and rabbit ARFGAP1 antibody. DAPI staining indicates the nucleus (blue).

To access the role of ARFGAP1 in the EV-A71 life cycle, we applied the siRNA strategy to silencing ARFGAP1 and used QS11 (an inhibitor of ARFGAP1) to disrupt COPI. The EV-A71 VP1 protein was decreased when ARFGAP1 was knocked down by two siRNA 3 days before EV-A71 infection (Figure 7A). ARFGAP1 depletion also reduced EV-A71 viral RNA replication assessed by QPCR (Figure 7B). However, silencing ARFGAP1 did not change the virus entry (Figure 7C). Furthermore, the EV-A71 virus titer was reduced by siRNA against ARFGAP1 (Figure 7D). Consistent with the proviral role of COPI in EV-A71 replication (Wang et al., 2012), we found that QS11 reduced EV-A71 replication while the cell viability was not changed by QS11 (Figure 7E,F). Altogether, ARFGAP1 promoted EV-A71 replication.

Next, we examined the localization of ARFGAP1 in EV-A71 infected RD cells. As shown in Figure 7G, ARFGAP1 partially colocalized with viral dsRNA, further indicating that ARFGAP1 might promote viral replication.

## Discussion

EV-A71 replication relies on many host factors (Shih et al., 2011). Identifying novel viral host factors will help us better understand EV-A71 biology. In the present study, we applied GST pull-down or GFP-Trap immunoprecipitation coupled with mass spectrometry to identify the cellular interactome of EV-A71. Similar strategies have been applied to identify the host interactomes for other viruses (Gao et al., 2017; Hafirassou et al., 2017; Li et al., 2017; Coyaud et al., 2018; Wang et al., 2018). We demonstrated that the EV-A71 2C protein interacted with TRIM4, exportin2, and ARFGAP1. TRIM4, exportin2, and ARFGAP1 are required for EV-A71 replication. Moreover, exportin2 and ARFGAP1 also interact with 2C proteins encoded by other enteroviruses. Further characterization of the other hits identified in this study may be beneficial for a better understanding of the biology of EV-A71 and 2C.

We have previously performed yeast 2 hybrid unbiased screenings to identify 2C binding partners, and several important regulators including p65 have been identified (Du et al., 2015). Others have identified more 2C-associated host proteins including reticulon3, coatomer, IKKβ and protein phosphatase 1 (Tang et al., 2007; Zheng et al., 2011; Wang et al., 2012; Li et al., 2016). Three 2C-associated host proteins (hnRNPK, COPB2, and PKM) were on the list of our MS SPEC results, indicating the validity of our screen. Some of the previously identified factors (reticulon3, p65, protein phosphatase 1) were not on our screening list and we identified several novel 2C associate factors, including TRIM4, exportin2, and ARFGAP1. The differences can be explained by the specific features of the individual screening methods. The design of this study is the use of the GST fusion protein or GFP-2C coupled with MS SPEC. This may help identify transcription related factors, which are normally excluded in the Y2H screen.

In this study, we found that TRIM4 is required for EV-A71 replication. As a member of the TRIM family, TRIM4 contains 500 amino acids, including three zinc-binding domains, one RING-finger domain, one type 1 B-box, one type 2 B-box and one coiled-coil domain. TRIM4 is localized in the cytoplasmic body and is widely expressed in many tissues and cells, but its function is poorly understood (Tomar et al., 2015). Recently, studies have shown that TRIM4 can regulate the K63 ubiquitination of the Retinoic Acid-Inducible Gene 1 Protein (RIG-I) and the assembly of mitochondrial antiviral signal complexes (Yan et al., 2014). Other studies have shown that TRIM4 plays a role in the regulation of oxidative stress induced cell death by interacting with peroxiredoxin 1 (PRX1) (Tomar et al., 2015). Upon EV-A71 infection, TRIM4 localized with viral dsRNA. TRIM4 might be involved in EV-A71 replication organelle formation by association with 2C. The other possibility is that TRIM4 regulates mitochondrial ROS generation (Tomar et al., 2015), which is critical for EV71 infection. Future studies need to be done to dissect the detailed mechanism of how TRIM4 promotes EV-A71 replication.

Some proteins with nuclear localization sequences need cofactors to transport to the nucleus, and importin α/β heterodimers play an important role in this process (Goldfarb et al., 2004; Kimura and Imamoto, 2014). Importin α binds to nuclear localization sequences, while importin β regulates transport through nuclear pore complexes. When proteins are transported to the nucleus, they are widely distributed in the nucleus, whereas RanGTP binds to importin β and replaces importin α. Importin α must return to the cytoplasm, and the protein that binds to it remains in the nucleus to complete the transport process. The transport of importin α from nucleus to cytoplasm is regulated by exportin2, also named the chromosome segregation 1-like protein (CSE1L) and the cellular apoptosis susceptibility protein (CAS) (Behrens et al., 2003). Only in the presence of RanGTP can exportin2 bind tightly with importin α to form an importin α/exportin2/RanGTP complex (Tanaka et al., 2007; Jiang, 2016). Importin α is released in the cytoplasm and interacts with RanBP1 and RanGAP1. The identification of exportin2 as a positive regulator for EV-A71 replication is interesting. Exportin2 is a multi-functional protein that plays a role in apoptosis, chromosome assembly during mitosis, cellular proliferation, microvesicles formation, and nucleocytoplasmic transport (Bera et al., 2001; Tanaka et al., 2007; Jiang, 2016). Exportin2 shuttles the importins from the nucleus to the cytoplasm, where importins deliver transcription factors to the nucleus (Stewart, 2007). We speculated that exportin2 possibly functions by facilitating the nuclear import of certain transcription factors transcribing the proviral host factors for EV-A71.

In eukaryotic cells, the transport of intracellular cargo is mainly accomplished by coated vesicles, such as COPI, COPII and the clathrin vesicle. COPI coat, which mediates vesicle transport from Golgi to endoplasmic reticulum (ER), is composed of coatomer, its accessory proteins and cargoes (Hsu and Yang, 2009). The accessory proteins in COPI are ADP Ribosylation Factor 1 (ARF1), Golgi Brefeldin A Resistant Guanine Nucleotide Exchange Factor 1 (GBFl), ARFGAP1 (Hsu and Yang, 2009; Hsu, 2011). There are two forms of ARF1: GTP-ARF1 and GDP-ARF1. ARF1 is activated from GBF1 to GTP-ARF1, while ARFGAP1 can turn ARF1 into GDP form (Hsu and Yang, 2009). In addition, studies have shown that ARFGAP1 is a component of the COPI vesicle and also plays a cargo sorting role in COPI vesicle formation (Yang et al., 2002; Lee et al., 2005). Identifying the proviral role of ARFGAP1 further reinforces the critical role of COPI in EV-A71 replication. Previous studies suggests that key components of COPI, including coatomer and ARF1, are host factors of EV-A71 (Wang et al., 2012, 2014). Previously, ARFGAP1 was also participated in the replication of hepatitis C virus replication (Li et al., 2014). We speculated that EV-A71 2C recruited ARFGAP1 to the viral replication area through protein-protein interaction. ARFGAP1 together with other COPI components promoted EV-A71 replication.

## Conclusion

In summary, we elucidated the host interactome of EV-A71 2C. Functional characterization of selected interaction partners *in vitro* revealed three host dependency factors including TRIM4, exportin2, and ARFGAP1 for EV-A71 replication. Our proteomic results provide a data-rich resource for the study of EV-71 in general. The host dependency factors we identified may contribute to the development of novel antiviral therapeutic avenues.

## Author Contributions

YL and XJ performed the majority of the experiments with help from PY. GZ offered technical assistance. YL and LZ analyzed the data. LZ conceived the research and wrote the manuscript.

## Conflict of Interest Statement

The authors declare that the research was conducted in the absence of any commercial or financial relationships that could be construed as a potential conflict of interest.
