# Supplementary material for: Elucidating the Host Interactome of EV-A71 2C Reveals Viral Dependency Factors
Source: Front Microbiol. 2019 Apr 2;10:636. doi: 10.3389/fmicb.2019.00636 (PMC6454016; doi:10.3389/fmicb.2019.00636)
Supplement: Supplementary file 1 [file Table_1.docx]

**Supplementary Tables**

Supplementary Table 1. GST pulldown/MS screens for cellular interaction partners of GST-2C.

| number | protein name | protein description |
| --- | --- | --- |
| 1 | CBR1 | Carbonyl reductase [NADPH] 1 |
| 2 | EEF1A1P5 | Putative elongation factor 1-alpha-like 3 |
| 3 | EEF1A2 | Elongation factor 1-alpha 2 |
| 4 | HIST1H4A | Histone H4 |
| 5 | HIST2H2BF | Histone H2B type 2-F |
| 6 | EEF1G | Elongation factor 1-gamma |
| 7 | ACTA1 | Actin, alpha skeletal muscle |
| 8 | EEF1B2 | Elongation factor 1-beta |
| 9 | LDHA | L-lactate dehydrogenase A chain |
| 10 | RPS3 | 40S ribosomal protein S3 |
| 11 | HSPA8 | Heat shock cognate 71 kDa protein |
| 12 | HIST1H2AG | Histone H2A type 1 |
| 13 | ANKHD1 | Ankyrin repeat and KH domain-containing protein 1 |
| 14 | PDZK1 | Na(+)/H(+) exchange regulatory cofactor NHE-RF3 |
| 15 | ALB | Serum albumin |
| 16 | RPS10 | 40S ribosomal protein S10 |
| 17 | EPHA8 | Ephrin type-A receptor 8 |
| 18 | MYH13 | Myosin-13 |
| 19 | HIST2H3A | Histone H3.2 |
| 20 | CTTNBP2NL | CTTNBP2 N-terminal-like protein |
| 21 | RPS16 | 40S ribosomal protein S16 |
| 22 | RPS18 | 40S ribosomal protein S18 |
| 23 | HIST1H1D | Histone H1.3 |
| 24 | PRDX1 | Peroxiredoxin-1 |
| 25 | UNC80 | Protein unc-80 homolog |
| 26 | MYH4 | Myosin-4 |
| 27 | TRGV3 | T-cell receptor gamma chain V region PT-gamma-1/2 |
| 28 | OBSL1 | Obscurin-like protein 1 |
| 29 | RAB1B | Ras-related protein Rab-1B |
| 30 | NPM1 | Nucleophosmin |
| 31 | RAB43 | Ras-related protein Rab-43 |
| 32 | MYH1 | Myosin-1 |
| 33 | MUC16 | Mucin-16 |
| 34 | RAB3C | Ras-related protein Rab-3C |
| 35 | RAB3A | Ras-related protein Rab-3A |
| 36 | RPL8 | 60S ribosomal protein L8 |
| 37 | PYGM | Glycogen phosphorylase, muscle form |
| 38 | PUS10 | Putative tRNA pseudouridine synthase Pus10 |
| 39 | RPL14 | 60S ribosomal protein L14 |
| 40 | PHACTR3 | Phosphatase and actin regulator 3 |
| 41 | HSP90AA1 | Heat shock protein HSP 90-alpha |
| 42 | CCDC63 | Coiled-coil domain-containing protein 63 |
| 43 | SYNE1 | Nesprin-1 OS=Homo sapiens |
| 44 | PPM1J | Protein phosphatase 1J |
| 45 | TRIM4 | Tripartite motif-containing protein 4 |
| 46 | RAB33B | Ras-related protein Rab-33B |
| 47 | RAB6B | Ras-related protein Rab-6B |
| 48 | RAB15 | Ras-related protein Rab-15 |
| 49 | PIP | Prolactin-inducible protein |
| 50 | ARAP2 | Arf-GAP with Rho-GAP domain, ANK repeat and PH domain-containing protein 2 |
| 51 | UCKL1 | Uridine-cytidine kinase-like 1 |
| 52 | ASAP2 | Arf-GAP with SH3 domain, ANK repeat and PH domain-containing protein 2 |
| 53 | ACLY | ATP-citrate synthase |
| 54 | HSD17B1 | Estradiol 17-beta-dehydrogenase 1 |
| 55 | DIAPH2 | Protein diaphanous homolog 2 |
| 56 | PRKDC | DNA-dependent protein kinase catalytic subunit |
| 57 | TTN | Titin |
| 58 | RPS5 | 40S ribosomal protein S5 |
| 59 | UHRF1BP1L | UHRF1-binding protein 1-like |
| 60 | POLDIP3 | Polymerase delta-interacting protein 3 |
| 61 | RPS9 | 40S ribosomal protein S9 |
| 62 | HSPB1 | Heat shock protein beta-1 |
| 63 | CCDC37 | Coiled-coil domain-containing protein 37 |
| 64 | LAMA2 | Laminin subunit alpha-2 |
| 65 | CYP26B1 | Cytochrome P450 26B1 |
| 66 | PCLO | Protein piccolo |
| 67 | MORC4 | MORC family CW-type zinc finger protein 4 |
| 68 | NEK10 | Serine/threonine-protein kinase Nek10 |
| 69 | LINC00479 | Putative uncharacterized protein encoded by LINC00479 |
| 70 | XPO2 | Exportin2 |
| 71 | SSC4D | Scavenger receptor cysteine-rich domain-containing group B protein O |
| 72 | LRGUK | Leucine-rich repeat and guanylate kinase domain-containing protein |
| 73 | NFATC2 | Nuclear factor of activated T-cells, cytoplasmic 2 |
| 74 | COQ6 | Ubiquinone biosynthesis monooxygenase COQ6 |

Supplementary Table 2. GST pulldown/MS screens for cellular interaction partners of GST-2C(126-263).

| number | protein name | protein description |
| --- | --- | --- |
| 1 | CBR1 | Carbonyl reductase [NADPH] 1 |
| 2 | CBR3 | Carbonyl reductase [NADPH] 3 |
| 3 | EEF1A1 | Elongation factor 1-alpha 1 |
| 4 | CTTNBP2NL | CTTNBP2 N-terminal-like protein |
| 5 | HSPA1A | Heat shock 70 kDa protein 1A |
| 6 | HSPA1L | Heat shock 70 kDa protein 1-like |
| 7 | HSPA6 | Heat shock 70 kDa protein 6 |
| 8 | EEF1B2 | Elongation factor 1-beta |
| 9 | ALB | Serum albumin |
| 10 | HSPA8 | Heat shock cognate 71 kDa protein |
| 11 | EEF1G | Elongation factor 1-gamma |
| 12 | HIST1H4A | Histone H4 |
| 13 | PRDX1 | Peroxiredoxin-1 |
| 14 | SFMBT2 | Scm-like with four MBT domains protein 2 |
| 15 | NCF2 | Neutrophil cytosol factor 2 |
| 16 | ANKHD1 | Ankyrin repeat and KH domain-containing protein 1 |
| 17 | CDHR2 | Cadherin-related family member 2 |
| 18 | HSPA9 | Stress-70 protein, mitochondrial |
| 19 | IMPA1 | Inositol monophosphatase 1 |
| 20 | RPL38 | 60S ribosomal protein L38 |
| 21 | GRPEL1 | GrpE protein homolog 1, mitochondrial |
| 22 | ATP5J2 | ATP synthase subunit f, mitochondrial |
| 23 | ASAP2 | Arf-GAP with SH3 domain, ANK repeat and PH domain-containing protein 2 |
| 24 | C10orf12 | Uncharacterized protein C10orf12 |
| 25 | PKD1L3 | Polycystic kidney disease protein 1-like 3 |
| 26 | XAGE1A | X antigen family member 1 |
| 27 | PRPF40A | Pre-mRNA-processing factor 40 homolog A |
| 28 | TRIM4 | E3 ubiquitin-protein ligase TRIM4 |
| 29 | VIM | Vimentin |
| 30 | SERPINE2 | Glia-derived nexin |
| 31 | RDH12 | Retinol dehydrogenase 12 |
| 32 | HIST2H2BF | Histone H2B type 2-F |
| 33 | ATP5A1 | ATP synthase subunit alpha, mitochondrial |
| 34 | OR52Z1 | Olfactory receptor 52Z1 |
| 35 | HRNR | Hornerin |
| 36 | NEK10 | Serine/threonine-protein kinase Nek10 |
| 37 | LINC00479 | Putative uncharacterized protein encoded by LINC00479 |
| 38 | XPO2 | Exportin2 |
| 39 | SPTBN2 | Spectrin beta chain, non-erythrocytic 2 |

Supplementary Table 3. GST pulldown/MS screens for cellular interaction partners of GST-2C(264-329).

| number | protein name | protein description |
| --- | --- | --- |
| 1 | CTTNBP2NL | CTTNBP2 N-terminal-like protein |
| 2 | ALB | Serum albumin |
| 3 | SFMBT2 | Scm-like with four MBT domains protein 2 |
| 4 | CDHR2 | Cadherin-related family member 2 |
| 5 | EEF1A1P5 | Putative elongation factor 1-alpha-like 3 |
| 6 | PRDX1 | Peroxiredoxin-1 |
| 7 | EEF1G | Elongation factor 1-gamma |
| 8 | GFAP | Glial fibrillary acidic protein |
| 9 | HSPA1A | Heat shock 70 kDa protein 1A |
| 10 | HSPA1L | Heat shock 70 kDa protein 1-like |
| 11 | PPM1G | Protein phosphatase 1G |
| 12 | HSPA6 | Heat shock 70 kDa protein 6 |
| 13 | PRDX2 | Peroxiredoxin-2 |
| 14 | ANKHD1 | Ankyrin repeat and KH domain-containing protein 1 |
| 15 | HSPA8 | Heat shock cognate 71 kDa protein |
| 16 | HSPA2 | Heat shock-related 70 kDa protein 2 |
| 17 | NRROS | Negative regulator of reactive oxygen species |
| 18 | ASAP2 | Arf-GAP with SH3 domain, ANK repeat and PH domain-containing protein 2 |
| 19 | HRNR | Hornerin |
| 20 | UNC79 | Protein unc-79 homolog |
| 21 | HSPA9 | Stress-70 protein, mitochondrial |
| 22 | ROCK2 | Rho-associated protein kinase 2 |
| 23 | ADAM7 | Disintegrin and metalloproteinase domain-containing protein 7 |
| 24 | ATP7A | Copper-transporting ATPase 1 |

Supplementary Table 4. GFP-Trap immunoprecipitation /MS screens for cellular interaction partners of GFP-2C.

| number | protein name | protein description |
| --- | --- | --- |
| 1 | ACTG1 | Actin, cytoplasmic 2 |
| 2 | TMPO | Lamina-associated polypeptide 2, isoforms beta/gamma |
| 3 | RPS27A | Ubiquitin-40S ribosomal protein S27a |
| 4 | HSPA1A | Heat shock 70 kDa protein 1A |
| 5 | HSPD1 | 60 kDa heat shock protein, mitochondrial |
| 6 | VIM | Vimentin |
| 7 | HSPA8 | Heat shock cognate 71 kDa protein |
| 8 | ATP5A1 | ATP synthase subunit alpha, mitochondrial |
| 9 | NPM1 | Nucleophosmin |
| 10 | NCL | Nucleolin |
| 11 | CSRP2 | Cysteine and glycine-rich protein 2 |
| 12 | ATP5B | ATP synthase subunit beta, mitochondrial |
| 13 | EEF1A1P5 | Putative elongation factor 1-alpha-like 3 |
| 14 | POTEF | POTE ankyrin domain family member F |
| 15 | MIF | Macrophage migration inhibitory factor |
| 16 | PHGDH | D-3-phosphoglycerate dehydrogenase |
| 17 | ACTBL2 | Beta-actin-like protein 2 |
| 18 | CBS | Cystathionine beta-synthase |
| 19 | KHSRP | Far upstream element-binding protein 2 |
| 20 | SERBP1 | Plasminogen activator inhibitor 1 RNA-binding protein |
| 21 | PGRMC1 | Membrane-associated progesterone receptor component 1 |
| 22 | ATP1A1 | Sodium/potassium-transporting ATPase subunit alpha-1 |
| 23 | HSPA9 | Stress-70 protein, mitochondrial |
| 24 | CTPS1 | CTP synthase 1 |
| 25 | RCC2 | Protein RCC2 |
| 26 | RPL4 | 60S ribosomal protein L4 |
| 27 | EEF2 | Elongation factor 2 |
| 28 | RPS19 | 40S ribosomal protein S19 |
| 29 | PRDX1 | Peroxiredoxin-1 |
| 30 | PHB | Prohibitin |
| 31 | HSPA6 | Heat shock 70 kDa protein 6 |
| 32 | PHB2 | Prohibitin-2 |
| 33 | XPO2 | Exportin2 |
| 34 | SLC25A3 | Phosphate carrier protein, mitochondrial |
| 35 | HNRNPA1 | Heterogeneous nuclear ribonucleoprotein A1 |
| 36 | SFPQ | Splicing factor, proline- and glutamine-rich |
| 37 | RPL6 | 60S ribosomal protein L6 |
| 38 | HSP90AB1 | Heat shock protein HSP 90-beta |
| 39 | PARK7 | Protein deglycase DJ-1 |
| 40 | CANX | Calnexin |
| 41 | HSP90AA1 | Heat shock protein HSP 90-alpha |
| 42 | ATP5O | ATP synthase subunit O, mitochondrial |
| 43 | HIST1H2AG | Histone H2A type 1 |
| 44 | HIST1H2BK | Histone H2B type 1-K |
| 45 | HNRNPM | Heterogeneous nuclear ribonucleoprotein M |
| 46 | RPL7A | 60S ribosomal protein L7a |
| 47 | PROSC | Proline synthase co-transcribed bacterial homolog protein |
| 48 | STOML2 | Stomatin-like protein 2, mitochondrial |
| 49 | RPN1 | Dolichyl-diphosphooligosaccharide--protein glycosyltransferase subunit 1 |
| 50 | PARP1 | Poly [ADP-ribose] polymerase 1 |
| 51 | RPS3A | 40S ribosomal protein S3a |
| 52 | IRS4 | Insulin receptor substrate 4 |
| 53 | CRKL | Crk-like protein |
| 54 | PABPC1 | Polyadenylate-binding protein 1 |
| 55 | RPL12 | 60S ribosomal protein L12 |
| 56 | CORO1C | Coronin-1C |
| 57 | FASN | Fatty acid synthase |
| 58 | RPS5 | 40S ribosomal protein S5 |
| 59 | RPL24 | 60S ribosomal protein L24 |
| 60 | IPO5 | Importin-5 |
| 61 | RAB5C | Ras-related protein Rab-5C |
| 62 | LRPPRC | Leucine-rich PPR motif-containing protein, mitochondrial |
| 63 | RPL3 | 60S ribosomal protein L3 |
| 64 | SLC25A5 | ADP/ATP translocase 2 |
| 65 | RPL26 | 60S ribosomal protein L26 |
| 66 | TARDBP | TAR DNA-binding protein 43 |
| 67 | PCBP1 | Poly(rC)-binding protein 1 |
| 68 | HIST1H1E | Histone H1.4 |
| 69 | UQCRC2 | Cytochrome b-c1 complex subunit 2, mitochondrial |
| 70 | HNRNPU | Heterogeneous nuclear ribonucleoprotein U |
| 71 | RPL19 | 60S ribosomal protein L19 |
| 72 | ALB | Serum albumin |
| 73 | SEC61B | Protein transport protein Sec61 subunit beta |
| 74 | CFL1 | Cofilin-1 |
| 75 | CLTC | Clathrin heavy chain 1 |
| 76 | EMD | Emerin |
| 77 | PPIA | Peptidyl-prolyl cis-trans isomerase A |
| 78 | FN3KRP | Ketosamine-3-kinase |
| 79 | RPS27 | 40S ribosomal protein S27 |
| 80 | EEF1G | Elongation factor 1-gamma |
| 81 | NOLC1 | Nucleolar and coiled-body phosphoprotein 1 |
| 82 | PCMT1 | Protein-L-isoaspartate(D-aspartate) O-methyltransferase |
| 83 | PCBP2 | Poly(rC)-binding protein 2 |
| 84 | GART | Trifunctional purine biosynthetic protein adenosine-3 |
| 85 | RPL7 | 60S ribosomal protein L7 |
| 86 | RPL13 | 60S ribosomal protein L13 |
| 87 | IMMT | MICOS complex subunit MIC60 |
| 88 | RPS2 | 40S ribosomal protein S2 |
| 89 | RPS18 | 40S ribosomal protein S18 |
| 90 | HSPA5 | 78 kDa glucose-regulated protein |
| 91 | RPS11 | 40S ribosomal protein S11 |
| 92 | PFN2 | Profilin-2 |
| 93 | LRRC59 | Leucine-rich repeat-containing protein 59 |
| 94 | HNRNPA2B1 | Heterogeneous nuclear ribonucleoproteins A2/B1 |
| 95 | RPS14 | 40S ribosomal protein S14 |
| 96 | SLC16A1 | Monocarboxylate transporter 1 |
| 97 | MAP4 | Microtubule-associated protein 4 |
| 98 | RPL29 | 60S ribosomal protein L29 |
| 99 | EPRS | Bifunctional glutamate/proline--tRNA ligase |
| 100 | RPS6 | 40S ribosomal protein S6 |
| 101 | FAM217B | Protein FAM217B |
| 102 | DBN1 | Drebrin |
| 103 | LSM12 | Protein LSM12 homolog |
| 104 | TFRC | Transferrin receptor protein 1 |
| 105 | UBA1 | Ubiquitin-like modifier-activating enzyme 1 |
| 106 | RPL8 | 60S ribosomal protein L8 |
| 107 | RPS25 | 40S ribosomal protein S25 |
| 108 | NONO | Non-POU domain-containing octamer-binding protein |
| 109 | SLC25A6 | ADP/ATP translocase 3 |
| 110 | NTPCR | Cancer-related nucleoside-triphosphatase |
| 111 | ATP2A2 | Sarcoplasmic/endoplasmic reticulum calcium ATPase 2 |
| 112 | RPS20 | 40S ribosomal protein S20 |
| 113 | CAPZA1 | F-actin-capping protein subunit alpha-1 |
| 114 | RPL10A | 60S ribosomal protein L10a |
| 115 | RPS3 | 40S ribosomal protein S3 |
| 116 | LDHB | L-lactate dehydrogenase B chain |
| 117 | SLC3A2 | 4F2 cell-surface antigen heavy chain |
| 118 | RAB1BL | Ras-related protein Rap-1b-like protein |
| 119 | RPL23 | 60S ribosomal protein L23 |
| 120 | MTHFD1 | C-1-tetrahydrofolate synthase, cytoplasmic |
| 121 | GSR | Glutathione reductase, mitochondrial |
| 122 | RPS17L | 40S ribosomal protein S17-like |
| 123 | RPSA | 40S ribosomal protein SA |
| 124 | RPL5 | 60S ribosomal protein L5 |
| 125 | HIST1H4A | Histone H4 |
| 126 | RAB1B | Ras-related protein Rab-1B |
| 127 | SLC1A5 | Neutral amino acid transporter B(0) |
| 128 | UQCRC1 | Cytochrome b-c1 complex subunit 1, mitochondrial |
| 129 | RPL23A | 60S ribosomal protein L23a |
| 130 | DNAJC10 | DnaJ homolog subfamily C member 10 |
| 131 | CCT7 | T-complex protein 1 subunit eta |
| 132 | TXN | Thioredoxin |
| 133 | OPA1 | Dynamin-like 120 kDa protein, mitochondrial |
| 134 | SCAMP3 | Secretory carrier-associated membrane protein 3 |
| 135 | TRAP1 | Heat shock protein 75 kDa, mitochondrial |
| 136 | SEC22B | Vesicle-trafficking protein SEC22b |
| 137 | RAP1A | Ras-related protein Rap-1A |
| 138 | HNRNPH1 | Heterogeneous nuclear ribonucleoprotein H |
| 139 | ATP1B3 | Sodium/potassium-transporting ATPase subunit beta-3 |
| 140 | DNAJA1 | DnaJ homolog subfamily A member 1 |
| 141 | KPNB1 | Importin subunit beta-1 |
| 142 | RPS29 | 40S ribosomal protein S29 |
| 143 | CCT8 | T-complex protein 1 subunit theta |
| 144 | RBM4 | RNA-binding protein 4 |
| 145 | TMEM109 | Transmembrane protein 109 |
| 146 | DUT | Deoxyuridine 5~-triphosphate nucleotidohydrolase, mitochondrial |
| 147 | PKM | Pyruvate kinase PKM |
| 148 | PABPC4 | Polyadenylate-binding protein 4 |
| 149 | TNPO1 | Transportin-1 |
| 150 | TIMM44 | Mitochondrial import inner membrane translocase subunit TIM44 OS=Homo sapiens |
| 151 | HDAC6 | Histone deacetylase 6 |
| 152 | PTBP1 | Polypyrimidine tract-binding protein 1 |
| 153 | RHOA | Transforming protein RhoA |
| 154 | DHX9 | ATP-dependent RNA helicase A |
| 155 | RPL27A | 60S ribosomal protein L27a |
| 156 | RPL11 | 60S ribosomal protein L11 |
| 157 | ATAD3B | ATPase family AAA domain-containing protein 3B |
| 158 | RPL17 | 60S ribosomal protein L17 |
| 159 | CCT4 | T-complex protein 1 subunit delta |
| 160 | DSG2 | Desmoglein-2 |
| 161 | BOLA2 | BolA-like protein 2 |
| 162 | GANAB | Neutral alpha-glucosidase AB |
| 163 | HNRNPA0 | Heterogeneous nuclear ribonucleoprotein A0 |
| 164 | SRPRB | Signal recognition particle receptor subunit beta |
| 165 | RPS7 | 40S ribosomal protein S7 |
| 166 | PRDX2 | Peroxiredoxin-2 |
| 167 | RPS4X | 40S ribosomal protein S4, X isoform |
| 168 | MYO1B | Unconventional myosin-Ib |
| 169 | RAB10 | Ras-related protein Rab-10 |
| 170 | RPL38 | 60S ribosomal protein L38 |
| 171 | COMT | Catechol O-methyltransferase |
| 172 | RPS8 | 40S ribosomal protein S8 |
| 173 | RPL18 | 60S ribosomal protein L18 |
| 174 | VAT1 | Synaptic vesicle membrane protein VAT-1 homolog |
| 175 | PGRMC2 | Membrane-associated progesterone receptor component 2 |
| 176 | TMX1 | Thioredoxin-related transmembrane protein 1 |
| 177 | MT-CO2 | Cytochrome c oxidase subunit 2 |
| 178 | GAPDH | Glyceraldehyde-3-phosphate dehydrogenase |
| 179 | VDAC1 | Voltage-dependent anion-selective channel protein 1 |
| 180 | PLS3 | Plastin-3 |
| 181 | CD19 | B-lymphocyte antigen CD19 |
| 182 | GSPT2 | Eukaryotic peptide chain release factor GTP-binding subunit ERF3B |
| 183 | ZC3HAV1L | Zinc finger CCCH-type antiviral protein 1-like |
| 184 | ILF3 | Interleukin enhancer-binding factor 3 |
| 185 | RPL31 | 60S ribosomal protein L31 |
| 186 | RPL21 | 60S ribosomal protein L21 |
| 187 | ENO1 | Alpha-enolase |
| 188 | GNB1L | Guanine nucleotide-binding protein subunit beta-like protein 1 |
| 189 | RAB7A | Ras-related protein Rab-7a |
| 190 | RAB1A | Ras-related protein Rab-1A |
| 191 | MAT2A | S-adenosylmethionine synthase isoform type-2 |
| 192 | HNRNPAB | Heterogeneous nuclear ribonucleoprotein A/B |
| 193 | SRP14 | Signal recognition particle 14 kDa protein |
| 194 | YBX1 | Nuclease-sensitive element-binding protein 1 |
| 195 | CCT3 | T-complex protein 1 subunit gamma |
| 196 | XPO1 | Exportin1 |
| 197 | LYN | Tyrosine-protein kinase Lyn |
| 198 | HADHB | Trifunctional enzyme subunit beta, mitochondrial |
| 199 | TCP1 | T-complex protein 1 subunit alpha |
| 200 | HADHA | Trifunctional enzyme subunit alpha, mitochondrial |
| 201 | RPS16 | 40S ribosomal protein S16 |
| 202 | PRSS1 | Trypsin-1 |
| 203 | FARSB | Phenylalanine--tRNA ligase beta subunit |
| 204 | TOMM70A | Mitochondrial import receptor subunit TOM70 |
| 205 | DDOST | Dolichyl-diphosphooligosaccharide--protein glycosyltransferase 48 kDa subunit |
| 206 | RTFDC1 | Protein RTF2 homolog |
| 207 | BSG | Basigin |
| 208 | SR4 | Translocon-associated protein subunit delta |
| 209 | RAB14 | Ras-related protein Rab-14 |
| 210 | NOTCH3 | Neurogenic locus notch homolog protein 3 |
| 211 | SMN1 | Survival motor neuron protein |
| 212 | HCCS | Cytochrome c-type heme lyase |
| 213 | LDHA | L-lactate dehydrogenase A chain |
| 214 | MRPS27 | 28S ribosomal protein S27, mitochondrial |
| 215 | LCK | Tyrosine-protein kinase Lck |
| 216 | RBMX | RNA-binding motif protein, X chromosome |
| 217 | RAN | GTP-binding nuclear protein Ran |
| 218 | SRSF6 | Serine/arginine-rich splicing factor 6 |
| 219 | RPS10 | 40S ribosomal protein S10 |
| 220 | RPL36A | 60S ribosomal protein L36a |
| 221 | MARCKSL1 | MARCKS-related protein |
| 222 | TOR1AIP1 | Torsin-1A-interacting protein 1 |
| 223 | GNB1 | Guanine nucleotide-binding protein G(I)/G(S)/G(T) subunit beta-1 |
| 224 | ATP12A | Potassium-transporting ATPase alpha chain 2 |
| 225 | SLIRP | SRA stem-loop-interacting RNA-binding protein, mitochondrial |
| 226 | EIF5AL1 | Eukaryotic translation initiation factor 5A-1-like |
| 227 | RUVBL1 | RuvB-like 1 |
| 228 | COX4I1 | Cytochrome c oxidase subunit 4 isoform 1, mitochondrial |
| 229 | SEC31B | Protein transport protein Sec31B |
| 230 | NSDHL | Sterol-4-alpha-carboxylate 3-dehydrogenase, decarboxylating |
| 231 | ZW10 | Centromere/kinetochore protein zw10 homolog |
| 232 | GNB2 | Guanine nucleotide-binding protein G(I)/G(S)/G(T) subunit beta-2 |
| 233 | CISD2 | CDGSH iron-sulfur domain-containing protein 2 |
| 234 | RPL10L | 60S ribosomal protein L10-like |
| 235 | CCT6A | T-complex protein 1 subunit zeta |
| 236 | SCO1 | Protein SCO1 homolog, mitochondrial |
| 237 | VCP | Transitional endoplasmic reticulum ATPase |
| 238 | NEK10 | Serine/threonine-protein kinase Nek10 |
| 239 | SRSF3 | Serine/arginine-rich splicing factor 3 |
| 240 | PLS1 | Plastin-1 |
| 241 | RPL13A | 60S ribosomal protein L13a |
| 242 | SH3GL1 | Endophilin-A2 |
| 243 | UQCRFS1P1 | Putative cytochrome b-c1 complex subunit Rieske-like protein 1 |
| 244 | MRPL39 | 39S ribosomal protein L39, mitochondrial |
| 245 | PGAM5 | Serine/threonine-protein phosphatase PGAM5, mitochondrial |
| 246 | EHMT1 | Histone-lysine N-methyltransferase EHMT1 |
| 247 | LAMTOR1 | Ragulator complex protein LAMTOR1 |
| 248 | PSMD2 | 26S proteasome non-ATPase regulatory subunit 2 |
| 249 | NDUFS3 | NADH dehydrogenase [ubiquinone] iron-sulfur protein 3, mitochondrial |
| 250 | RPS9 | 40S ribosomal protein S9 |
| 251 | HNRNPA3 | Heterogeneous nuclear ribonucleoprotein A3 |
| 252 | ISOC2 | Isochorismatase domain-containing protein 2, mitochondrial |
| 253 | HLA-A | HLA class I histocompatibility antigen, A-2 alpha chain |
| 254 | TOMM22 | Mitochondrial import receptor subunit TOM22 homolog |
| 255 | PRDX6 | Peroxiredoxin-6 |
| 256 | HSDL2 | Hydroxysteroid dehydrogenase-like protein 2 |
| 257 | DHX15 | Pre-mRNA-splicing factor ATP-dependent RNA helicase DHX15 |
| 258 | ESYT1 | Extended synaptotagmin-1 |
| 259 | CD81 | CD81 antigen |
| 260 | DDX17 | Probable ATP-dependent RNA helicase DDX17 |
| 261 | ADRM1 | Proteasomal ubiquitin receptor ADRM1 |
| 262 | ATP5I | ATP synthase subunit e, mitochondrial |
| 263 | MYO1C | Unconventional myosin-Ic |
| 264 | NME1 | Nucleoside diphosphate kinase A |
| 265 | UBE2M | NEDD8-conjugating enzyme Ubc12 |
| 266 | NDUFAF2 | Mimitin, mitochondrial |
| 267 | PDCD6 | Programmed cell death protein 6 |
| 268 | DDRGK1 | DDRGK domain-containing protein 1 |
| 269 | ISOC1 | Isochorismatase domain-containing protein 1 |
| 270 | HSPH1 | Heat shock protein 105 kDa |
| 271 | CAPZB | F-actin-capping protein subunit beta |
| 272 | RPL14 | 60S ribosomal protein L14 |
| 273 | KARS | Lysine--tRNA ligase |
| 274 | NIPSNAP1 | Protein NipSnap homolog 1 |
| 275 | TIMM50 | Mitochondrial import inner membrane translocase subunit TIM50 |
| 276 | RPL27 | 60S ribosomal protein L27 |
| 277 | RUVBL2 | RuvB-like 2 |
| 278 | GMPS | GMP synthase [glutamine-hydrolyzing] |
| 279 | SSBP1 | Single-stranded DNA-binding protein, mitochondrial |
| 280 | TP53 | Cellular tumor antigen p53 |
| 281 | NDUFA10 | NADH dehydrogenase [ubiquinone] 1 alpha subcomplex subunit 10, mitochondrial |
| 282 | WDR6 | WD repeat-containing protein 6 |
| 283 | C1QBP | Complement component 1 Q subcomponent-binding protein, mitochondrial |
| 284 | MARCKS | Myristoylated alanine-rich C-kinase substrate |
| 285 | MCM5 | DNA replication licensing factor MCM5 |
| 286 | TTC1 | Tetratricopeptide repeat protein 1 |
| 287 | CYB5R3 | NADH-cytochrome b5 reductase 3 |
| 288 | NUP155 | Nuclear pore complex protein Nup155 |
| 289 | PSMD9 | 26S proteasome non-ATPase regulatory subunit 9 |
| 290 | CYC1 | Cytochrome c1, heme protein, mitochondrial |
| 291 | CKAP5 | Cytoskeleton-associated protein 5 |
| 292 | XRCC5 | X-ray repair cross-complementing protein 5 |
| 293 | VBP1 | Prefoldin subunit 3 |
| 294 | PRKAR2A | cAMP-dependent protein kinase type II-alpha regulatory subunit O |
| 295 | BA57 | Putative transferase CAF17, mitochondrial |
| 296 | TOMM20 | Mitochondrial import receptor subunit TOM20 homolog |
| 297 | MCMBP | Mini-chromosome maintenance complex-binding protein |
| 298 | VDAC2 | Voltage-dependent anion-selective channel protein 2 |
| 299 | JUP | Junction plakoglobin |
| 300 | TUFM | Elongation factor Tu, mitochondrial |
| 301 | CCT2 | T-complex protein 1 subunit beta |
| 302 | DARS | Aspartate--tRNA ligase, cytoplasmic |
| 303 | DDX3X | ATP-dependent RNA helicase DDX3X |
| 304 | RPL36 | 60S ribosomal protein L36 |
| 305 | PITHD1 | PITH domain-containing protein 1 |
| 306 | GIPR | Gastric inhibitory polypeptide receptor |
| 307 | TARS2 | Threonine--tRNA ligase, mitochondrial |
| 308 | KATNAL2 | Katanin p60 ATPase-containing subunit A-like 2 |
| 309 | PSMF1 | Proteasome inhibitor PI31 subunit |
| 310 | BZW2 | Basic leucine zipper and W2 domain-containing protein 2 |
| 311 | MTDH | Protein LYRIC |
| 312 | CNP | 2~,3~-cyclic-nucleotide 3~-phosphodiesterase |
| 313 | GOLGB1 | Golgin subfamily B member 1 |
| 314 | RPL18A | 60S ribosomal protein L18a |
| 315 | HIST2H3A | Histone H3.2 |
| 316 | DNASE2B | Deoxyribonuclease-2-beta |
| 317 | RPS13 | 40S ribosomal protein S13 |
| 318 | DDX21 | Nucleolar RNA helicase 2 |
| 319 | MCCC2 | Methylcrotonoyl-CoA carboxylase beta chain, mitochondrial |
| 320 | RPL35 | 60S ribosomal protein L35 |
| 321 | EPHA8 | Ephrin type-A receptor 8 |
| 322 | PL15 | 60S ribosomal protein L15 |
| 323 | VPS35 | Vacuolar protein sorting-associated protein 35 |
| 324 | C4orf22 | Uncharacterized protein C4orf22 |
| 325 | RPL37 | 60S ribosomal protein L37 |
| 326 | NFKBIE | NF-kappa-B inhibitor epsilon |
| 327 | MTHFD1L | Monofunctional C1-tetrahydrofolate synthase, mitochondrial |
| 328 | HTRA2 | Serine protease HTRA2, mitochondrial |
| 329 | PDHB | Pyruvate dehydrogenase E1 component subunit beta, mitochondrial |
| 330 | DCTPP1 | dCTP pyrophosphatase 1 |
| 331 | RAB8A | Ras-related protein Rab-8A |
| 332 | HSP90B1 | Endoplasmin |
| 333 | MAT2B | Methionine adenosyltransferase 2 subunit beta |
| 334 | SLC7A5 | Large neutral amino acids transporter small subunit 1 |
| 335 | ZMPSTE24 | CAAX prenyl protease 1 homolog |
| 336 | AMKMT | Calmodulin-lysine N-methyltransferase |
| 337 | DYNC1I2 | Cytoplasmic dynein 1 intermediate chain 2 |
| 338 | HSD17B10 | 3-hydroxyacyl-CoA dehydrogenase type-2 |
| 339 | TMEM245 | Transmembrane protein 245 |
| 340 | CHCHD3 | MICOS complex subunit MIC19 |
| 341 | PSMD7 | 26S proteasome non-ATPase regulatory subunit 7 |
| 342 | HRNR | Hornerin |
| 343 | USP5 | Ubiquitin carboxyl-terminal hydrolase 5 |
| 344 | C19orf43 | Uncharacterized protein C19orf43 |
| 345 | NDUFB4 | NADH dehydrogenase [ubiquinone] 1 beta subcomplex subunit 4 |
| 346 | NEK9 | Serine/threonine-protein kinase Nek9 |
| 347 | PCYT1A | Choline-phosphate cytidylyltransferase A |
| 348 | PPA2 | Inorganic pyrophosphatase 2, mitochondrial |
| 349 | XRN2 | 5~-3~ exoribonuclease 2 |
| 350 | CADM1 | Cell adhesion molecule 1 |
| 351 | NDUFA7 | NADH dehydrogenase [ubiquinone] 1 alpha subcomplex subunit 7 |
| 352 | ALDH18A1 | Delta-1-pyrroline-5-carboxylate synthase |
| 353 | NCAPH | Condensin complex subunit 2 |
| 354 | SEC61A1 | Protein transport protein Sec61 subunit alpha isoform 1 |
| 355 | QKI | Protein quaking |
| 356 | CSDE1 | Cold shock domain-containing protein E1 |
| 357 | AASDHPPT | L-aminoadipate-semialdehyde dehydrogenase-phosphopantetheinyl transferase |
| 358 | CPOX | Oxygen-dependent coproporphyrinogen-III oxidase, mitochondrial |
| 359 | ANXA2 | Annexin A2 |
| 360 | SDHA | Succinate dehydrogenase [ubiquinone] flavoprotein subunit, mitochondrial |
| 361 | NCAPD2 | Condensin complex subunit 1 |
| 362 | MATR3 | Matrin-3 |
| 363 | HLTF | Helicase-like transcription factor |
| 364 | CHCHD2P9 | Putative coiled-coil-helix-coiled-coil-helix domain-containing protein CHCHD2P9, mitochondrial |
| 365 | AGK | Acylglycerol kinase, mitochondrial |
| 366 | CYP51A1 | Lanosterol 14-alpha demethylase |
| 367 | DNAAF5 | Dynein assembly factor 5, axonemal |
| 368 | HNRNPK | Heterogeneous nuclear ribonucleoprotein K |
| 369 | MYO6 | Unconventional myosin-VI |
| 370 | DLST | Dihydrolipoyllysine-residue succinyltransferase component of 2-oxoglutarate dehydrogenase complex, mitochondrial |
| 371 | NDUFS7 | NADH dehydrogenase [ubiquinone] iron-sulfur protein 7, mitochondrial |
| 372 | RPLP0P6 | 60S acidic ribosomal protein P0-like |
| 373 | NDUFV2 | NADH dehydrogenase [ubiquinone] flavoprotein 2, mitochondrial |
| 374 | CD99 | CD99 antigen |
| 375 | SSR3 | Translocon-associated protein subunit gamma |
| 376 | ADO | 2-aminoethanethiol dioxygenase |
| 377 | AFG3L2 | AFG3-like protein 2 |
| 378 | CHEK1 | Serine/threonine-protein kinase Chk1 |
| 379 | ACLY | ATP-citrate synthase |
| 380 | AGTRAP | Type-1 angiotensin II receptor-associated protein |
| 381 | SMC4 | Structural maintenance of chromosomes protein 4 |
| 382 | GNAS | Guanine nucleotide-binding protein G(s) subunit alpha isoforms XLas |
| 383 | GNA12 | Guanine nucleotide-binding protein subunit alpha-12 |
| 384 | SCCPDH | Saccharopine dehydrogenase-like oxidoreductase |
| 385 | ATP2B2 | Plasma membrane calcium-transporting ATPase 2 |
| 386 | RPS26P11 | Putative 40S ribosomal protein S26-like 1 |
| 387 | CDC42 | Cell division control protein 42 homolog |
| 388 | SLC7A1 | High affinity cationic amino acid transporter 1 |
| 389 | NSF | Vesicle-fusing ATPase |
| 390 | CNBP | Cellular nucleic acid-binding protein |
| 391 | SRPR | Signal recognition particle receptor subunit alpha |
| 392 | CCNB1 | G2/mitotic-specific cyclin-B1 |
| 393 | COPB2 | Coatomer subunit beta~ |
| 394 | RAB21 | Ras-related protein Rab-21 |
| 395 | CTTN | Src substrate cortactin |
| 396 | SEC62 | Translocation protein SEC62 |
| 397 | TEX264 | Testis-expressed sequence 264 protein |
| 398 | UBXN4 | UBX domain-containing protein 4 |
| 399 | FAF2 | FAS-associated factor 2 |
| 400 | SNRPA1 | U2 small nuclear ribonucleoprotein A~ |
| 401 | TBC1D4 | TBC1 domain family member 4 |
| 402 | FARSA | Phenylalanine--tRNA ligase alpha subunit |
| 403 | TK1 | Thymidine kinase, cytosolic |
| 404 | SEC23IP | SEC23-interacting protein |
| 405 | EWSR1 | RNA-binding protein EWS |
| 406 | IMPDH2 | Inosine-5~-monophosphate dehydrogenase 2 |
| 407 | ST13P4 | Putative protein FAM10A4 |
| 408 | AIP | AH receptor-interacting protein |
| 409 | USP30 | Ubiquitin carboxyl-terminal hydrolase 30 |
| 410 | GBAS | Protein NipSnap homolog 2 |
| 411 | SFXN1 | Sideroflexin-1 |
| 412 | AIMP2 | Aminoacyl tRNA synthase complex-interacting multifunctional protein 2 |
| 413 | SCRIB | Protein scribble homolog |
| 414 | DERL2 | Derlin-2 |
| 415 | RTCB | tRNA-splicing ligase RtcB homolog |
| 416 | HMGB1 | High mobility group protein B1 |
| 417 | SMC1A | Structural maintenance of chromosomes protein 1A |
| 418 | HNRNPF | Heterogeneous nuclear ribonucleoprotein F |
| 419 | MCM3 | DNA replication licensing factor MCM3 |
| 420 | MRPS5 | 28S ribosomal protein S5, mitochondrial |
| 421 | NOSIP | Nitric oxide synthase-interacting protein |
| 422 | CAPRIN1 | Caprin-1 |
| 423 | SEC63 | Translocation protein SEC63 homolog |
| 424 | UNC5D | Netrin receptor UNC5D |
| 425 | LINC00479 | Putative uncharacterized protein encoded by LINC00479 |
| 426 | PPP6C | Serine/threonine-protein phosphatase 6 catalytic subunit |
| 427 | LBR | Lamin-B receptor |
| 428 | DOCK2 | Dedicator of cytokinesis protein 2 |
| 429 | TECR | Very-long-chain enoyl-CoA reductase |
| 430 | OCIAD1 | OCIA domain-containing protein 1 |
| 431 | LIMA1 | LIM domain and actin-binding protein 1 |
| 432 | DNPEP | Aspartyl aminopeptidase |
| 433 | CEP170B | Centrosomal protein of 170 kDa protein B |
| 434 | CHMP1A | Charged multivesicular body protein 1a |
| 435 | MARS | Methionine--tRNA ligase, cytoplasmic |
| 436 | RAC2 | Ras-related C3 botulinum toxin substrate 2 |
| 437 | TRIM37 | E3 ubiquitin-protein ligase TRIM37 |
| 438 | ARMC1 | Armadillo repeat-containing protein 1 |
| 439 | TXLNG | Gamma-taxilin |
| 440 | TRIM65 | Tripartite motif-containing protein 65 |
| 441 | NDUFS2 | NADH dehydrogenase [ubiquinone] iron-sulfur protein 2, mitochondrial |
| 442 | YEATS4 | YEATS domain-containing protein 4 |
| 443 | HDGF | Hepatoma-derived growth factor |
| 444 | ARFGAP1 | ADP-ribosylation factor GTPase-activating protein 1 |
| 445 | IGF2BP1 | Insulin-like growth factor 2 mRNA-binding protein 1 |
| 446 | RRBP1 | Ribosome-binding protein 1 |
| 447 | FAM47B | Protein FAM47B |
| 448 | PTGES3 | Prostaglandin E synthase 3 |
| 449 | RPS23 | 40S ribosomal protein S23 |
| 450 | PTPN11 | Tyrosine-protein phosphatase non-receptor type 11 |
| 451 | NDUFC2-KCTD14 | NADH dehydrogenase [ubiquinone] 1 subunit C2, isoform 2 |
| 452 | AMOT | Angiomotin |
| 453 | LETM1 | LETM1 and EF-hand domain-containing protein 1, mitochondrial |
| 454 | DNAJC7 | DnaJ homolog subfamily C member 7 |
| 455 | FAU | 40S ribosomal protein S30 |
| 456 | DMBT1 | Deleted in malignant brain tumors 1 protein |
| 457 | LRRC40 | Leucine-rich repeat-containing protein 40 |
| 458 | ATXN2L | Ataxin-2-like protein |
| 459 | SACM1L | Phosphatidylinositide phosphatase SAC1 |
| 460 | SLC38A2 | Sodium-coupled neutral amino acid transporter 2 |
| 461 | PDHA1 | Pyruvate dehydrogenase E1 component subunit alpha, somatic form, mitochondrial |
| 462 | SDHB | Succinate dehydrogenase [ubiquinone] iron-sulfur subunit, mitochondrial |
| 463 | LRRC8E | Volume-regulated anion channel subunit LRRC8E |
| 464 | JAK3 | Tyrosine-protein kinase JAK3 |
| 465 | CTNND1 | Catenin delta-1 |
| 466 | CREBBP | CREB-binding protein |
| 467 | MYBBP1A | Myb-binding protein 1A |
| 468 | TNIK | TRAF2 and NCK-interacting protein kinase |
| 469 | AKAP1 | A-kinase anchor protein 1, mitochondrial |
| 470 | TRPC4AP | Short transient receptor potential channel 4-associated protein |
| 471 | CDK15 | Cyclin-dependent kinase 15 |
| 472 | TRIM28 | Transcription intermediary factor 1-beta |
| 473 | CLN6 | Ceroid-lipofuscinosis neuronal protein 6 |
| 474 | TCERG1 | Transcription elongation regulator 1 |
| 475 | MRPS34 | 28S ribosomal protein S34, mitochondrial |
| 476 | CALCA | Calcitonin |
| 477 | PUS10 | Putative tRNA pseudouridine synthase Pus10 |
| 478 | SPCS2 | Signal peptidase complex subunit 2 |
| 479 | SYAP1 | Synapse-associated protein 1 |
| 480 | XPO5 | Exportin5 |
| 481 | MRPS35 | 28S ribosomal protein S35, mitochondrial |
| 482 | DNAJA3 | DnaJ homolog subfamily A member 3, mitochondrial |
| 483 | XPNPEP3 | Probable Xaa-Pro aminopeptidase 3 |
| 484 | WIZ | Protein Wiz |
| 485 | XYLB | Xylulose kinase |
| 486 | VASH2 | Vasohibin-2 |
| 487 | UACA | Uveal autoantigen with coiled-coil domains and ankyrin repeats |
| 488 | SYNE2 | Nesprin-2 |
| 489 | GNMT | Glycine N-methyltransferase |
| 490 | DST | Dystonin |
| 491 | TNRC6B | Trinucleotide repeat-containing gene 6B protein |
| 492 | PITPNB | Phosphatidylinositol transfer protein beta isoform |
| 493 | NUMA1 | Nuclear mitotic apparatus protein 1 |
| 494 | RPL32 | 60S ribosomal protein L32 |
| 495 | GLYCTK | Glycerate kinase |
| 496 | ATP5F1 | ATP synthase F(0) complex subunit B1, mitochondrial |
| 497 | PLEKHH2 | Pleckstrin homology domain-containing family H member 2 |
| 498 | PABPC4L | Polyadenylate-binding protein 4-like |
| 499 | RPL39P5 | Putative 60S ribosomal protein L39-like 5 |
| 500 | LARS | Leucine--tRNA ligase, cytoplasmic |
| 501 | DHX30 | Putative ATP-dependent RNA helicase DHX30 |
| 502 | NME4 | Nucleoside diphosphate kinase, mitochondrial |
| 503 | EFHD2 | EF-hand domain-containing protein D2 |
| 504 | EEF1B2 | Elongation factor 1-beta |
